# Isolation and Genome Characterization of Escherichia Phage vB_EcoA-Sparklingdew

**DOI:** 10.3390/genes17060650

**Published:** 2026-05-31

**Authors:** Ivan M. Pchelin, Vladimir M. Shutov, T. N. Suong Nguyen, Dmitrii E. Polev, Alexander N. Suvorov, Artemiy E. Goncharov

**Affiliations:** 1 Department of Molecular Microbiology, Institute of Experimental Medicine, Saint Petersburg 197022, Russia; vladimir.shutov.2000y@mail.ru (V.M.S.); phage1@yandex.ru (A.E.G.); 2 Institute of Tropical Medicine, Joint Vietnam-Russia Tropical Science and Technology Research Center, Hanoi 100000, Vietnam; nguyenngocsuong641998@gmail.com; 3 St. Petersburg Pasteur Institute, Saint Petersburg 197101, Russia; 4 Department of Epidemiology, Parasitology and Disinfectology, North-Western State Medical University Named After I.I. Mechnikov, Saint Petersburg195067, Russia

**Keywords:** bacteriophages, *Kayfunavirus*, *Escherichia coli*, genome sequencing, repetitive sequences

## Abstract

Background: *Escherichia coli* remains a critical multidrug-resistant nosocomial pathogen, driving interest in bacteriophage-based biocontrol. The genus *Kayfunavirus* (family *Autotranscriptaviridae*) exhibits obligately lytic replication cycles and favorable biosafety profiles, yet each new phage requires comprehensive genomic characterization to expand therapeutic candidate pools. This study aimed to isolate and genomically characterize a novel *Kayfunavirus* from an environmental reservoir in Vietnam. Methods: Escherichia phage vB_EcoA-Sparklingdew was isolated from Can Tho River water using host *E. coli* AgE9. The genome was assembled using SPAdes. The termini were resolved with PhageTerm. The annotation was done via the Pharokka pipeline and HHpred. Taxonomic classification was performed using taxMyPhage, VIRIDIC intergenomic comparisons, and maximum likelihood phylogeny of concatenated structural proteins. Results: The complete genome comprises a 37,944 bp linear dsDNA molecule (49.9% GC), encoding 51 open reading frames in a predominantly unidirectional arrangement. Key features include a virion-encoded T7-like RNA polymerase, a 723-residue T7-like DNA polymerase, a canonical lysis triad, and two putative tailspike proteins. A 212 bp direct terminal repeat and coverage profiles support a headful (pac) packaging mechanism. Comprehensive screening confirmed the absence of lysogeny, virulence, and antibiotic resistance determinants. A single synonymous SNP indicated high clonal purity. Intergenomic identity peaked at 87.7% against ICTV references, confirming placement in a novel species. Conclusions: Phage Sparklingdew represents a strictly lytic *Kayfunavirus* with a compact genomic architecture. Its favorable safety profile and absence of temperate markers support further evaluation for targeted therapeutic applications against pathogenic *E. coli*.

## 1. Introduction

*Escherichia coli* is a prevalent nosocomial pathogen capable of developing antibiotic resistance and forming biofilms, substantiating the need for alternative therapeutic agents, including bacteriophages [1,2,3]. The global decline in antibiotic discovery and increasing multidrug resistance have led to renewed interest in phage therapy as a viable alternative for treating antibiotic-resistant bacterial infections [4,5,6]. Consequently, the systematic isolation and complete genomic annotation of novel phages are essential for expanding the phage therapeutic arsenal and elucidating phage–host coevolutionary dynamics.

Within the diverse landscape of tailed bacteriophages, the genus *Kayfunavirus* (family *Autotranscriptaviridae*, subfamily *Studiervirinae*) has attracted increasing attention due to its obligately lytic replication cycle, high host specificity, and favorable biosafety profile. Genomic studies reveal that *Kayfunavirus* members possess compact, linear double-stranded DNA genomes ranging from approximately 37.2 to 41.4 kbp, with GC content values varying between 49.7% and 53.2% [7,8,9]. These genomes generally encode 42 to 55 open reading frames (ORFs) organized into conserved functional modules. A key component of the replication and transcription module is a T3/T7-like RNA polymerase, a taxonomic hallmark of the class *Autographivirales*. The other gene modules encode functions related to virion morphogenesis, DNA packaging, and host cell lysis. Comprehensive screening consistently demonstrates the absence of virulence factors, antibiotic resistance genes, and lysogeny-associated integrases or recombinases across characterized isolates, reinforcing their suitability for clinical, veterinary, and food safety applications [7,8,10]. Furthermore, recent studies highlight a predominantly unidirectional gene arrangement, coordinated by phage- and host-derived promoters and Rho-independent terminators, which streamlines transcriptional regulation during the rapid infection cycle [11].

A defining structural feature of many *Autographivirales* members, including *Kayfunavirus*, is the presence of terminal redundancies in their linear genomes. Direct terminal repeats (DTRs) are employed in genome packaging, often serving as recognition signals for the terminase complex or facilitating circular permutation and concatemer resolution during headful packaging. While DTRs are a recognized genomic trait within the genus, their length exhibits notable strain-specific variation: characterized isolates harbor DTRs of 179–286 bp [11,12,13,14]. Beyond terminal structures, internal repetitive DNA elements play roles in phage genome plasticity, homologous recombination, and evolutionary adaptation. Recent comparative analyses have uncovered extensive recombination landscapes in *Kayfunavirus* genomes, with hotspots frequently localized to tail fiber/adhesion regions, suggesting that repeat-mediated genetic exchange drives host range diversification and ecological adaptation [9,15].

The continuous discovery of novel *Kayfunavirus* strains, coupled with high-resolution genomic annotation, is essential to decipher genotype–phenotype correlations, refine taxonomic boundaries, and identify candidates for targeted biocontrol. In this study, we report the isolation and complete genome sequence characterization of Escherichia phage vB_EcoA-Sparklingdew, belonging to a novel *Kayfunavirus* species.

## 2. Materials and Methods

### 2.1. Bacterial Strain and Culture Conditions

The host strain *E. coli.* AgE9 (genome sequence accession JBWXIS000000000) was grown in 20 mL volumes of Luria–Bertani (LB) broth medium (Amresco, Solon, OH, USA) at 37 °C for 18 h under shaking conditions at 180 rpm.

### 2.2. Bacteriophage Isolation

Bacteriophage vB_EcoA-Sparklingdew was isolated from Can Tho River (Can Tho, Vietnam, coordinates 10.031728 N, 105.788293 E) in October 2025. The 45 mL filtered water sample (using a 0.22 μm pore-size PVDF membrane syringe filter) was mixed with 5 mL 10× TSB broth and 200 μL 18 h bacterial culture. Upon 18 h incubation and filtration, the presence of bacteriophages was tested in spot tests on bacterial lawns. To ensure clonal purity, three consecutive rounds of single plaque isolation were performed by picking a single plaque with a sterile pipette tip and resuspending it in 1 mL LB broth. The suspension was added to 10 mL of exponentially growing *E. coli* culture to the optical density measured at 600 nm (OD_600_) ≈ 0.5, incubated statically for 18 h at 37 °C, and centrifuged in a CM-6M benchtop centrifuge (ELMI, Riga, Latvia) at 1690× *g* for 20 min at 25 °C. The supernatant was filtered through a 0.22 μm pore-size PVDF membrane syringe filter to remove bacterial debris. Phage titer was quantified using the double-layer agar assay with 1.5% bottom LB agar and 0.7% overlay agar containing 200 μL of bacterial culture at 10^9^ CFU/mL and 1 mL serially diluted phage suspension. Phage lysates were stored at 4 °C. The third passage preparation was subjected to DNA isolation and genome sequencing.

### 2.3. Whole Genome Sequencing and Assembly

Bacteriophage DNA isolation and purification were performed using the Cells, Tissues, Blood DNA Extraction Spin Column Kit (Biolabmix, Novosibirsk, Russia), employing doubled incubation times with proteinase K treatment as compared to the manufacturer’s protocol, with an actual duration of the step of 30 min. DNA content was measured with a Qubit Flex fluorometer (Thermo Fisher Scientific, Waltham, MA, USA) using a QuDye dsDNA HS Assay Kit (Lumiprobe RUS, Moscow, Russia). The DNA libraries were prepared using a IGT Enzyme Plus V3 library preparation kit (iGeneTech, Beijing, China) and sequenced on a DNBSEQ-G50 sequencer (MGI, Shenzhen, China) in PE150 mode. Read quality control and adapter removal were done with Trim Galore v0.6.10 (https://github.com/FelixKrueger/TrimGalore, accessed on 18 February 2026). The genome reads were assembled in SPAdes v4.2.0 assembler [16] using the --meta option.

### 2.4. Genome Annotation

Bacteriophage genome ends were identified with PhageTerm v4.2 [17]. A search for nucleotide sequence repeats was carried out with the aid of MEME Suite v5.5.9 [18] with the "any number of repetitions" option, allowing motifs to occur zero or more times per sequence. The tool searched for a maximum of 3 motifs 6–50 bp wide. The site strand handling setting allowed sites on either DNA strand.

Open reading frame annotation was performed using the Pharokka pipeline, utilizing PHANOTATE v1.6.7 [19] for ab initio gene prediction. Coding sequences (CDSs) were identified using the HHPred webserver [20,21]. Hits with probability values ≥ 98% were considered significant. Functional annotation was generated by matching each CDS to the PHROGs [22], VFDB [23], and CARD [24] databases. The search for CRISPR/Cas-related genes was done with CRISPR/Cas Finder v4.2.20 [25] implemented on Proksee webserver [26].

SPAdes-corrected paired-end reads were aligned to the phage Sparklingdew genome assembly using HISAT2 v2.2.2 [27] in the --no-spliced-alignment mode with the --very-sensitive option. The resulting SAM alignment file was sorted by genomic coordinates using SAMtools v1.23.1. Single-nucleotide polymorphisms (SNPs) were identified using the BCFtools v1.19 mpileup and call pipeline [28] with the maximum analyzed alignment coverage depth of 8000×. The multi-allelic caller (-m) was employed, and only variant positions were retained (-v). To ensure high-confidence calls, variants were filtered to retain only those with a Phred-scaled quality score ≥ 20 and read depth > 10 using the bcftools view command.

### 2.5. Taxonomic Analysis

Taxonomic identification was confirmed using taxMyPhage v0.3.6 [29]. Pairwise intergenomic distances were computed and visualized with the VIRIDIC v1.1 tool [30]. For the sequence similarity analysis, *Kayfunavirus* genome sequence accessions (*n* = 71) were retrieved from the MSL41.v1 virus metadata resource file (ICTV release 20 March 2026, https://ictv.global/vmr, accessed on 7 April 2026). *Kayfunavirus* protein phylogeny was inferred in the PhyML v3.1 program [31] implemented in SeaView v5.0.5 [32], visualized in MEGA v12.1.12 [33], and edited with Inkscape v1.4.3. Phylogenetic analysis employed the LG+G+I substitution model with 6 across-site variation categories. The substitution model and its parameters were determined by a model selection analysis in Mega. Bootstrap analysis was done with 1000 replicates.

## 3. Results

### 3.1. Bacteriophage Genome Assembly and Annotation

The genome of Escherichia phage vB_EcoA-Sparklingdew was assembled de novo as a single contig measuring 37,944 bp with 252× mean whole genome coverage. A total of 2,421,321 sequencing reads were generated, with 93% successfully mapping to the phage genome. The GC content of the genome was 49.9%. At two nucleotide positions 85 bp downstream of the 3′-end of the predicted 212 bp direct terminal repeat, the PhageTerm-corrected contig had an abrupt 12.7× local drop in read coverage depth. The residual coverage implied the actual presence of a PhageTerm-inferred sequence variant in the DNA sample. Nevertheless, we retained the initial SPAdes assembly.

**Figure 1:**
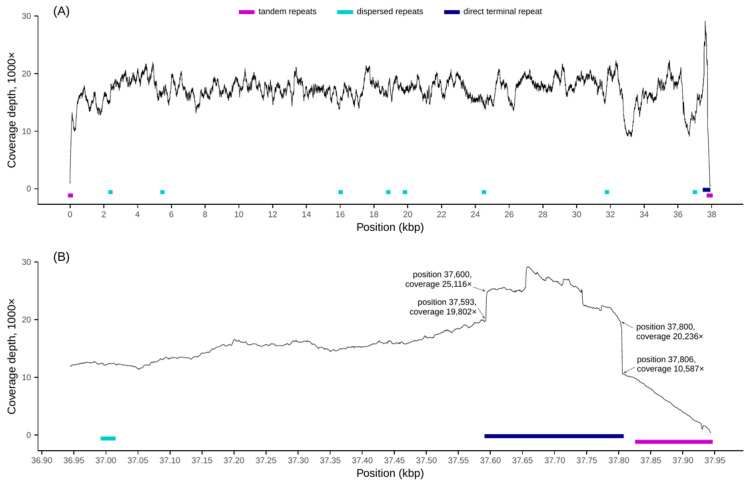
Escherichia phage vB_EcoA-Sparklingdew genome assembly. Sequence read coverage depth and nucleotide repeat loci. (**A**) The coverage graph spanning the whole assembly PZ247578.1. (**B**) 3′-end of the sequence spanning 1000 bp.

At both ends, the assembly had tandem repeat loci constituted by a 15 bp repeat unit 5′-AACCTACAGTCCTAC-3′ (Figure 1A). The direct terminal repeat predicted by PhageTerm was located at the 3′-end of the retained sequence with high read coverage (Figure 1B). Its 5′-end boundary was marked by a 1.27× coverage change, whereas the 3′-end co-located with a 1.91× coverage change. Coverage fluctuations at the DTR boundaries suggest sequence heterogeneity or packaging intermediates, though the core 212 bp repeat remains biologically relevant. Bacteriophage DNA packaging type prediction with the PhageTerm method suggested a headful (pac) mechanism. While Li’s method suggested a cos-type signature, likely driven by short terminal tandem repeats, the 212 bp DTR, terminal redundancy, and read coverage profile collectively support a headful (pac) packaging mechanism.

**Figure 2:**
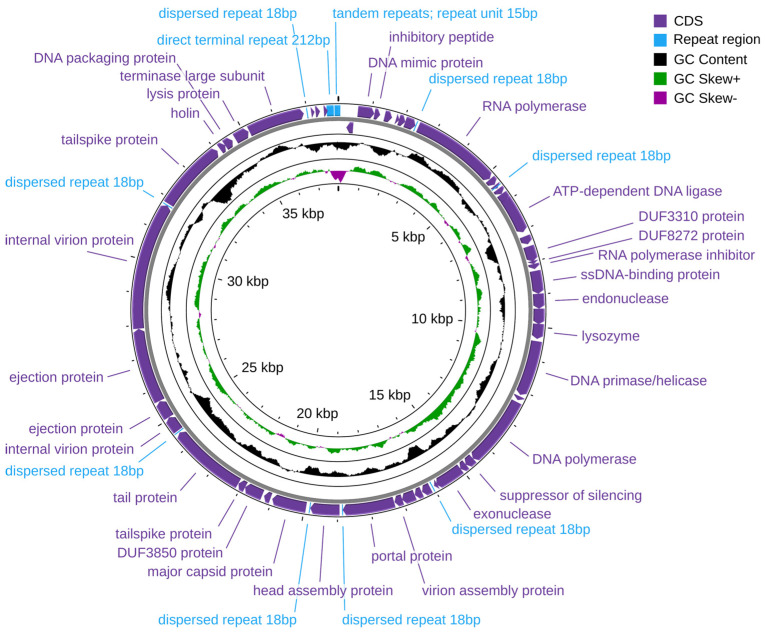
Escherichia phage vB_EcoA-Sparklingdew genome map prepared at the Proksee webserver. The linear assembly has identical tandem repeat loci at both ends.

Genomic annotation of this *Kayfunavirus* isolate revealed a compact, highly organized protein-coding repertoire comprising 51 open reading frames (ORFs, Figure 2). The ORFs were designated SPD_001 through SPD_051 in order of genomic position. Reflecting the predominantly unidirectional transcriptional architecture characteristic of the subfamily *Studiervirinae*, all protein-coding genes are arranged on the plus strand with the sole exception of the hypothetical protein gene SPD_001, which is oriented on the complementary strand. The genome is partitioned into conserved functional modules governing nucleic acid metabolism, virion morphogenesis, DNA packaging, host cell lysis, and host takeover.

Central to the replication and transcription module is the virion-encoded single-subunit RNA polymerase SPD_008, a definitive taxonomic hallmark of the class *Autographivirales*. This module is further represented by a T7-like DNA polymerase (SPD_024; 723 aa), whose length is consistent with the canonical length of similar *Studiervirinae* enzymes [34], alongside a DNA primase/helicase SPD_022, ATP-dependent DNA ligase SPD_013, exonuclease SPD_027, single-stranded DNA-binding protein SPD_018, and an endonuclease SPD_019.

Structural and packaging machinery is represented by a major capsid protein SPD_035, portal protein SPD_033, head assembly protein SPD_034, internal virion proteins SPD_040 and SPD_043, DNA ejection proteins SPD_041 and SPD_042, DNA packaging protein SPD_046, and a terminase large subunit SPD_048. The virion tail apparatus includes two distinct tailspike proteins, SPD_037 and SPD_044, and auxiliary tail component SPD_039, while host cell lysis is orchestrated by a canonical triad comprising a lysozyme SPD_021, a holin SPD_045, and an Rz-like lysis protein SPD_047. Host modulation is further mediated by auxiliary genes, such as a DNA mimic protein SPD_002, a direct inhibitor of bacterial cell division protein FtsZ SPD_003, an RNA polymerase inhibitor SPD_017, and a protein suppressor of silencing SPD_025. The remaining ORFs are annotated as hypothetical proteins or domains of unknown function, underscoring the specialized and rapidly evolving nature of phage genomic architecture. Consistent with the strictly lytic and biosafe profile of the genus, the screening confirmed the absence of coding sequences with integration or excision functions, virulence determinants, and antibiotic resistance genes. Additionally, CRISPR/Cas Finder analysis detected no CRISPR arrays or Cas genes.

The genome of phage Sparklingdew harbors eight dispersed 18 bp repeats with the consensus sequence 5′-CTWAACTATCACTATAGG-3′ (W = A/T). These elements are predominantly localized to the 3′-termini of coding sequences (SPD_007, SPD_033, SPD_034, and SPD_043) or within intergenic regions, with a single instance embedded internally within the hypothetical peptide gene SPD_010. The repeat length and characteristic core sequence TCACTATAGG imply that these are the promoter sites [35]. The single-nucleotide variant analysis revealed one point mutation. The T4673G substitution was located at the RNA polymerase gene SPD_008 and did not alter the predicted amino acid sequence, implying high homogeneity of the bacteriophage DNA sample.

### 3.2. Phage Identification and Taxonomic Classification

Taxonomic assignment using taxMyPhage confirmed that Escherichia phage vB_EcoA-Sparklingdew belongs to the genus *Kayfunavirus* (family *Autotranscriptaviridae*, subfamily *Studiervirinae*) and represents a previously undescribed species within this genus. Pairwise intergenomic similarity calculations against the ICTV MSL41.v1 reference dataset revealed nucleotide identity values ranging from 65.5% (accession MW258709.1) to 87.7% (accession OX460914.1) with characterized *Kayfunavirus* members (Figure S1). As current ICTV guidelines designate isolates sharing ≤ 95% genome-wide nucleotide identity as distinct species [36], these results support the classification of phage Sparklingdew as a representative of a novel species.

**Figure 3:**
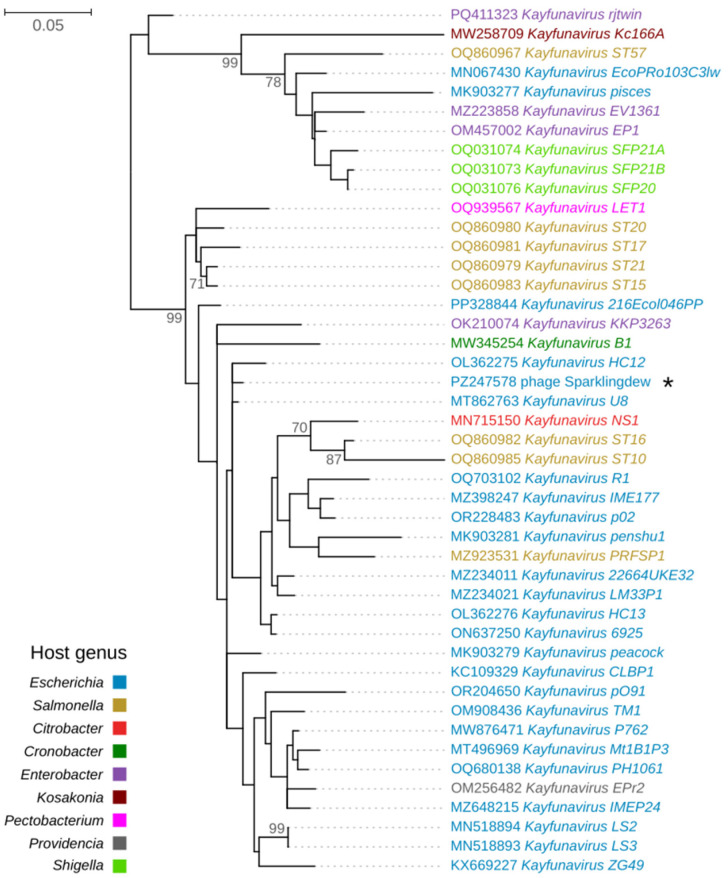
Maximum likelihood tree computed with concatenated major capsid protein and terminase large subunit amino acid sequences (*n* = 45). Bootstrap support values ≥ 70% are shown. The tree is rooted at the midpoint. Escherichia phage vB_EcoA-Sparklingdew is marked with an asterisk.

Phylogenetic reconstruction based on concatenated amino acid sequences of the major capsid protein and terminase large subunit further corroborated these findings. The maximum likelihood tree recovered two well-supported major clades within the genus (bootstrap support ≥ 70%); however, terminal branches exhibited limited stability under bootstrap resampling, reflecting the high sequence diversity and rapid evolutionary radiation characteristic of *Kayfunavirus* (Figure 3). Phage Sparklingdew occupied a distinct terminal position, consistent with its status as a representative of an uncharacterized species.

### 3.3. Genome Structural Conservation

**Figure 4:**
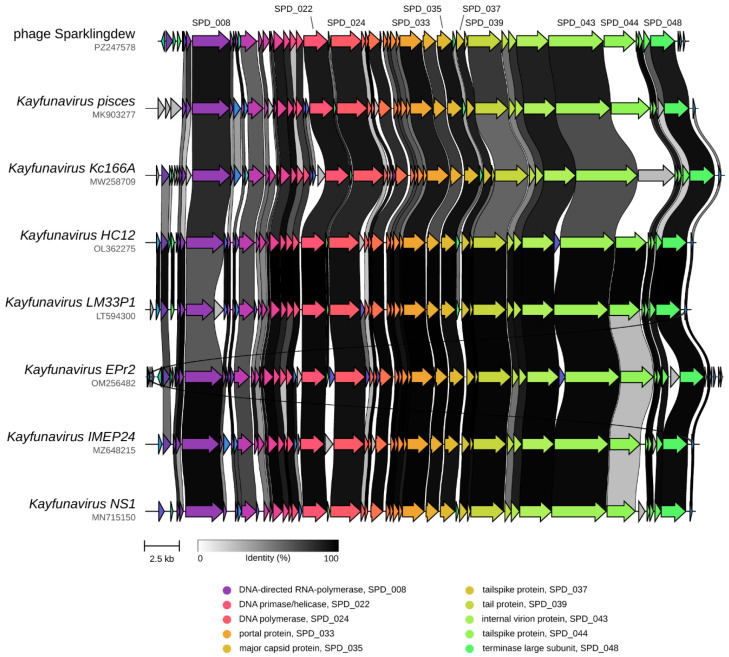
Gene conservation in *Kayfunavirus* visualized with clinker v0.0.32 [37] using the annotations from GenBank. The color codes relate to amino acid similarity sequence groups.

The *Kayfunavirus* members, including the novel phage Sparklingdew, exhibited the gene conservation patterns typical to members of a single strictly lytic phage genus, where the most protein-coding sequences are arranged in the same direction and share at least some degree of amino acid sequence identity. The tailspike protein SPD_044 was not conserved among all genomes (Figure 4). The other major components of the Sparklingdew genome functional modules were present across *Kayfunavirus* diversity at a moderate or high degree of sequence similarity. In the replication and transcription module, T7-like DNA polymerase and DNA primase/helicase were highly conserved. Being an example of a moderately conserved gene, the single-subunit RNA polymerase was identified as two separate enzymes in the Escherichia phage LM33_P1 genome (accession LT594300).

In the structural and packaging module, the same two types of sequences could be found, e.g., major capsid protein and terminase large subunit presented pairwise identity values close to 100%, and tail protein sequences (SPD_039) were variable. These observations indicate that *Kayfunavirus* genome evolution occurs on a scale of particular CDSs rather than whole gene modules.

## 4. Discussion

Escherichia phage vB_EcoA-Sparklingdew satisfies all genomic safety criteria relevant to therapeutic development. The genome lacks integrases, excisionases, virulence factors, and antibiotic resistance determinants, consistent with the obligately lytic lifestyle and favorable biosafety profile documented for *Kayfunavirus* isolates [12,13,38]. Functional annotation resolved a modular organization characteristic of the subfamily *Studiervirinae*: a replication module centered on a virion-encoded single-subunit RNA polymerase, structural genes for capsid assembly and DNA packaging, and a tripartite lysis cassette comprising holin, Rz-like protein, and lysozyme [11]. Single-nucleotide variant analysis identified only one high-confidence synonymous substitution (T4673G in SPD_008) across the 252× coverage assembly, confirming high clonal purity and minimal intra-lysate heterogeneity following plaque purification.

Taxonomic analyses place phage Sparklingdew within a novel species of *Kayfunavirus*. Pairwise intergenomic distances fall below the 95% nucleotide identity threshold used by ICTV to delineate species, and phylogenetic reconstruction using conserved structural proteins recovers phage Sparklingdew on a distinct terminal branch. The genome encodes two putative tailspike proteins, a configuration less common in T7-like podoviruses but observed in phages infecting Gram-negative hosts with complex surface polysaccharides. Experimental evolution studies demonstrate that coevolution between bacteria and phages drives accelerated, divergent molecular evolution specifically in genes governing host attachment, including tail fiber and structural adhesion proteins [39]. The overall genome architecture is compact and largely non-redundant outside of host interaction modules. The inferred presence of two distinct tailspike proteins should be experimentally validated. This dual-tailspike configuration is expected to enable adaptation to heterogeneous receptor landscapes or ongoing coevolutionary pressure [40], compensating for the limited genetic backup elsewhere in the genome.

Additional genomic features suggest mechanisms for host modulation and evolutionary flexibility. A protein suppressor of silencing, rare among tailed phages, may counteract small RNA-mediated bacterial immunity. Dispersed 18 bp repeats localized to 3′-ends of coding sequences or intergenic regions could facilitate recombination or transcriptional regulation, contributing to genomic plasticity [9]. Approximately half of the predicted proteins remain functionally uncharacterized, underscoring the still existing knowledge gaps and the potential for novel accessory functions.

In summary, Escherichia phage vB_EcoA-Sparklingdew is a strictly lytic member of the genus *Kayfunavirus*. It satisfies genomic requirements for a therapeutic bacteriophage, including the absence of lysogeny-associated genes, virulence factors, and antibiotic resistance determinants. The headful packaging mechanism with terminal redundancy is consistent with related phages in the genus. The favorable genomic properties support further characterization and potential development for therapeutic applications against pathogenic *E. coli* strains circulating in the Vietnamese population.

## Data Availability

The phage Sparklingdew genome sequence was deposited in the NCBI Nucleotide database with accession number PZ247578. The unassembled sequence reads were deposited with the SRA database accession SRX32935671.

## Supplementary Materials

The following supporting information can be downloaded at: https://www.mdpi.com/article/10.3390/genes17060650/s1, Figure S1: Heatmap of pairwise ANI values between phage Sparklingdew and ICTV-accepted *Kayfunavirus* members.

## References

[1] Paul M., Carrara E., Retamar P., Tängdén T., Bitterman R., Bonomo R.A., de Waele J., Daikos G.L., Akova M., Harbarth S. (2022). European Society of Clinical Microbiology and Infectious Diseases (ESCMID) guidelines for the treatment of infections caused by multidrug-resistant Gram-negative bacilli (endorsed by European society of intensive care medicine). Clin. Microbiol. Infect..

[2] Chegene Lorestani R., Shojaeian A., Rostamian M. (2023). Phenotypic, genotypic, and metabolic resistance mechanisms of ESKAPE bacteria to chemical disinfectants: A systematic review and meta-analysis. Expert. Rev. Anti Infect. Ther..

[3] WHO (2024). WHO Bacterial Priority Pathogens List, 2024.

[4] Nilsson A.S. (2019). Pharmacological limitations of phage therapy. Ups. J. Med. Sci..

[5] Stacey H.J., De Soir S., Jones J.D. (2022). The safety and efficacy of phage therapy: A systematic review of clinical and safety trials. Antibiotics.

[6] Kim M.K., Suh G.A., Cullen G.D., Perez Rodriguez S., Dharmaraj T., Chang T.H.W., Li Z., Chen Q., Green S.I., Lavigne R. (2025). Bacteriophage therapy for multidrug-resistant infections: Current technologies and therapeutic approaches. J. Clin. Investig..

[7] Petrzik K., Brázdová S., Krawczyk K. (2021). Novel viruses that lyse plant and human strains of *Kosakonia cowanii*. Viruses.

[8] Cao Y., Ma D., Zhou Y., Wang L., Han K., Li L., Mao X., Li Z., Wu Y., Liu H. (2023). Biological characteristics and genomic analysis of a novel *Escherichia* phage *Kayfunavirus CY1*. Virus Genes.

[9] Wan S., Li N., Li Y., Liang Y., Qu Y. (2026). Multidimensional characterization of a novel porcine *Klebsiella pneumoniae* phage Pkp-1. BMC Microbiol..

[10] Li L., Wu Y., Ma D., Zhou Y., Wang L., Han K., Cao Y., Wang X. (2022). Isolation and characterization of a novel *Escherichia coli* phage *Kayfunavirus ZH4*. Virus Genes.

[11] Vidigal P.M.P., Hungaro H.M. (2025). Genome sequencing of *Escherichia coli* phage UFJF_EcSW4 reveals a novel lytic *Kayfunavirus* species. 3 Biotech..

[12] Juliet R., Loganathan A., Neeravi A., Bakthavatchalam Y.D., Veeraraghavan B., Manohar P., Nachimuthu R. (2024). Characterization of *Salmonella* phage of the genus *Kayfunavirus* isolated from sewage infecting clinical strains of *Salmonella enterica*. Front. Microbiol..

[13] Yamaki S., Yamazaki K. (2024). Biological characterization and genomic analysis of a novel bacteriophage, MopsHU1, infecting *Morganella psychrotolerans*. Arch. Virol..

[14] Yuan X., Zhang S., Wang J., Li C., Li N., Yu S., Kong L., Zeng H., Yang G., Huang Y. (2021). Isolation and characterization of a novel *Escherichia coli Kayfunavirus* phage DY1. Virus Res..

[15] Tsai Y.C., Teh S.H., Huang P., Lin L.C., Lin N.T. (2025). Genomic, functional, and evolutionary insights into a novel T7-like phage B1 infecting multidrug-resistant *Enterobacter cloacae*. Int. J. Mol. Sci..

[16] Prjibelski A., Antipov D., Meleshko D., Lapidus A., Korobeynikov A. (2020). Using SPAdes de novo assembler. Curr. Protoc. Bioinform..

[17] Garneau J.R., Depardieu F., Fortier L.C., Bikard D., Monot M. (2017). PhageTerm: A tool for fast and accurate determination of phage termini and packaging mechanism using next-generation sequencing data. Sci. Rep..

[18] Bailey T.L., Johnson J., Grant C.E., Noble W.S. (2015). The MEME Suite. Nucleic Acids Res..

[19] McNair K., Zhou C., Dinsdale E.A., Souza B., Edwards R.A. (2019). PHANOTATE: A novel approach to gene identification in phage genomes. Bioinformatics.

[20] Zimmermann L., Stephens A., Nam S.Z., Rau D., Kübler J., Lozajic M., Gabler F., Söding J., Lupas A.N., Alva V. (2018). A completely reimplemented MPI bioinformatics toolkit with a new HHpred server at its core. J. Mol. Biol..

[21] Gabler F., Nam S.Z., Till S., Mirdita M., Steinegger M., Söding J., Lupas A.N., Alva V. (2020). Protein sequence analysis using the MPI bioinformatics toolkit. Curr. Protoc. Bioinform..

[22] Terzian P., Olo Ndela E., Galiez C., Lossouarn J., Pérez Bucio R.E., Mom R., Toussaint A., Petit M.A., Enault F. (2021). PHROG: Families of prokaryotic virus proteins clustered using remote homology. NAR. Genom. Bioinform..

[23] Chen L., Yang J., Yu J., Yao Z., Sun L., Shen Y., Jin Q. (2005). VFDB: A reference database for bacterial virulence factors. Nucleic Acids Res..

[24] Alcock B.P., Raphenya A.R., Lau T.T.Y., Tsang K.K., Bouchard M., Edalatmand A., Huynh W., Nguyen A.V., Cheng A.A., Liu S. (2020). CARD 2020: Antibiotic resistome surveillance with the comprehensive antibiotic resistance database. Nucleic Acids Res..

[25] Couvin D., Bernheim A., Toffano-Nioche C., Touchon M., Michalik J., Néron B., Rocha E.P.C., Vergnaud G., Gautheret D., Pourcel C. (2018). CRISPRCasFinder, an update of CRISRFinder, includes a portable version, enhanced performance and integrates search for Cas proteins. Nucleic Acids Res..

[26] Grant J.R., Enns E., Marinier E., Mandal A., Herman E.K., Chen C.Y., Graham M., Van Domselaar G., Stothard P. (2023). Proksee: In-depth characterization and visualization of bacterial genomes. Nucleic Acids Res..

[27] Kim D., Paggi J.M., Park C., Bennett C., Salzberg S.L. (2019). Graph-based genome alignment and genotyping with HISAT2 and HISAT-genotype. Nat. Biotechnol..

[28] Danecek P., Bonfield J.K., Liddle J., Marshall J., Ohan V., Pollard M.O., Whitwham A., Keane T., McCarthy S.A., Davies R.M. (2021). Twelve years of SAMtools and BCFtools. Gigascience.

[29] Millard A., Denise R., Lestido M., Nicholas M.T., Webster D., Turner D., Sicheritz-Pontén T. (2025). taxMyPhage: Automated taxonomy of dsDNA phage genomes at the genus and species level. PHAGE.

[30] Moraru C., Varsani A., Kropinski A.M. (2020). VIRIDIC-a novel tool to calculate the intergenomic similarities of prokaryote-infecting viruses. Viruses.

[31] Guindon S., Dufayard J.F., Lefort V., Anisimova M., Hordijk W., Gascuel O. (2010). New algorithms and methods to estimate maximum-likelihood phylogenies: Assessing the performance of PhyML 3.0. Syst. Biol..

[32] Gouy M., Guindon S., Gascuel O. (2010). SeaView version 4: A multiplatform graphical user interface for sequence alignment and phylogenetic tree building. Mol. Biol. Evol..

[33] Stecher G., Suleski M., Tao Q., Tamura K., Kumar S. (2026). MEGA 12.1: Cross-platform release for macOS and Linux operating systems. J. Mol. Evol..

[34] Dutta S., Li Y., Johnson D., Dzantiev L., Richardson C.C., Romano L.J., Ellenberger T. (2004). Crystal structures of 2-acetylaminofluorene and 2-aminofluorene in complex with T7 DNA polymerase reveal mechanisms of mutagenesis. Proc. Natl. Acad. Sci. USA.

[35] Chen Z., Schneider T.D. (2005). Information theory based T7-like promoter models: Classification of bacteriophages and differential evolution of promoters and their polymerases. Nucleic Acids Res..

[36] Turner D., Kropinski A.M., Adriaenssens E.M. (2021). A roadmap for genome-based phage taxonomy. Viruses.

[37] Gilchrist C.L.M., Chooi Y.H. (2021). clinker & clustermap.js: Automatic generation of gene cluster comparison figures. Bioinformatics.

[38] Chaudhary N., Mohan B., Mavuduru R.S., Kumar Y., Taneja N. (2022). Characterization, genome analysis and in vitro activity of a novel phage vB_EcoA_RDN8.1 active against multi-drug resistant and extensively drug-resistant biofilm-forming uropathogenic *Escherichia coli* isolates, India. J. Appl. Microbiol..

[39] Paterson S., Vogwill T., Buckling A., Benmayor R., Spiers A.J., Thomson N.R., Quail M., Smith F., Walker D., Libberton B. (2010). Antagonistic coevolution accelerates molecular evolution. Nature.

[40] de Jonge P.A., Nobrega F.L., Brouns S.J.J., Dutilh B.E. (2019). Molecular and evolutionary determinants of bacteriophage host range. Trends Microbiol..

