# Supplementary figures and images for: Isolation and Genome Characterization of Escherichia Phage vB_EcoA-Sparklingdew
Source: Genes (Basel). 2026 May 31;17(6):650. doi: 10.3390/genes17060650 (PMC13298934; doi:10.3390/genes17060650)

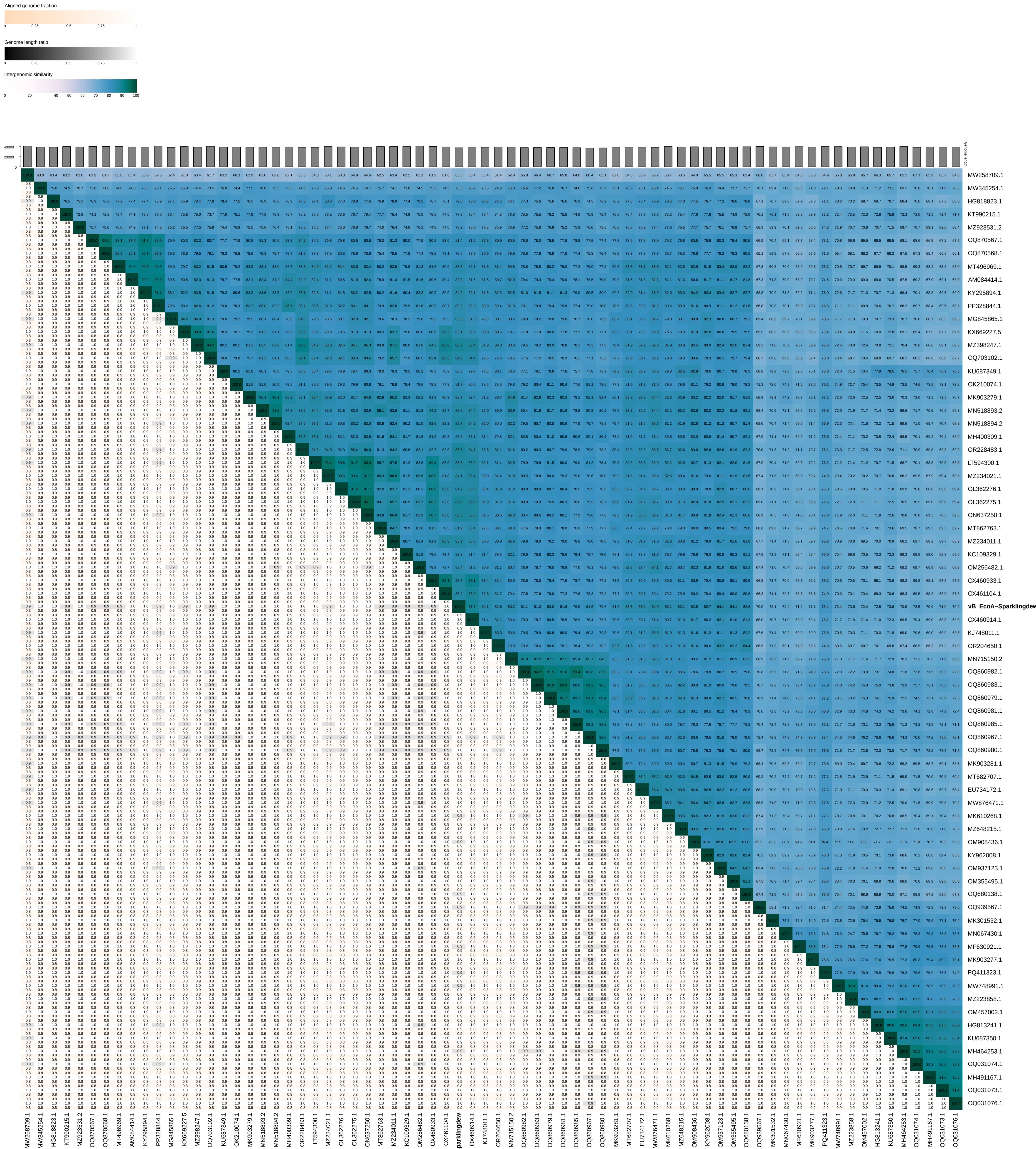

Supplement: Supplementary file 1 [file genes-17-00650-s001.zip › genes-4317142-Figure S1.pdf]
